# Genetic evidence against monophyly of Oniscidea implies a need to revise scenarios for the origin of terrestrial isopods

**DOI:** 10.1038/s41598-019-55071-4

**Published:** 2019-12-06

**Authors:** Andreas C. Dimitriou, Stefano Taiti, Spyros Sfenthourakis

**Affiliations:** 1 Department of Biological Sciences, University of Cyprus, Panepistimiou Ave. 1, 2109 Aglantzia, Nicosia Cyprus; 2 Museo di Storia Naturale, Sezione di Zoologia “La Specola”, Via Romana 17, 50125 Florence, Italy

**Keywords:** Molecular ecology, Phylogenetics

## Abstract

Among the few crustacean taxa that managed to inhabit terrestrial environments, Oniscidea includes the most successful colonizers in terms of species richness and abundance. However, neither morphological traits nor molecular markers have definitively resolved phylogenetic relationships among major Oniscidea clades or established the monophyly of the taxon. Herein, we employed the highly conserved, nuclear protein-coding genes Sodium-Potassium Pump (NAK) and Phosphoenolpyruvate Carboxykinase (PEPCK), along with the traditionally used 18 s and 28 s ribosomal RNA genes, in an attempt to clarify these questions. Our dataset included sequences representing all major Oniscidea clades and closely related aquatic taxa, as suggested by previous studies. We applied Bayesian Inference and Maximum Likelihood methods and produced a robust and fully resolved phylogenetic tree that offers strong evidence against the monophyly of Oniscidea. The amphibious genus *Ligia* appears to be more closely related to representatives of marine suborders, while the phylogenetic pattern of the remaining Oniscidea implies a complex history of the transition from the marine environment to land. With the exception of the basal clade, all other established major clades have been recovered as monophyletic, even though relationships within these clades call for a revised interpretation of morphological characters used in terrestrial isopod taxonomy.

## Introduction

Among the 11 suborders currently identified in Isopoda, Oniscidea is the only terrestrial suborder and by far the richest, comprising more than 3,700 described species^1,2^. Despite their generally limited dispersal abilities and their ancestors’ dependence on aquatic environments, they managed to extend their presence all over the globe and inhabit most types of habitats, including deserts^2–4^.

**Figure 1:**
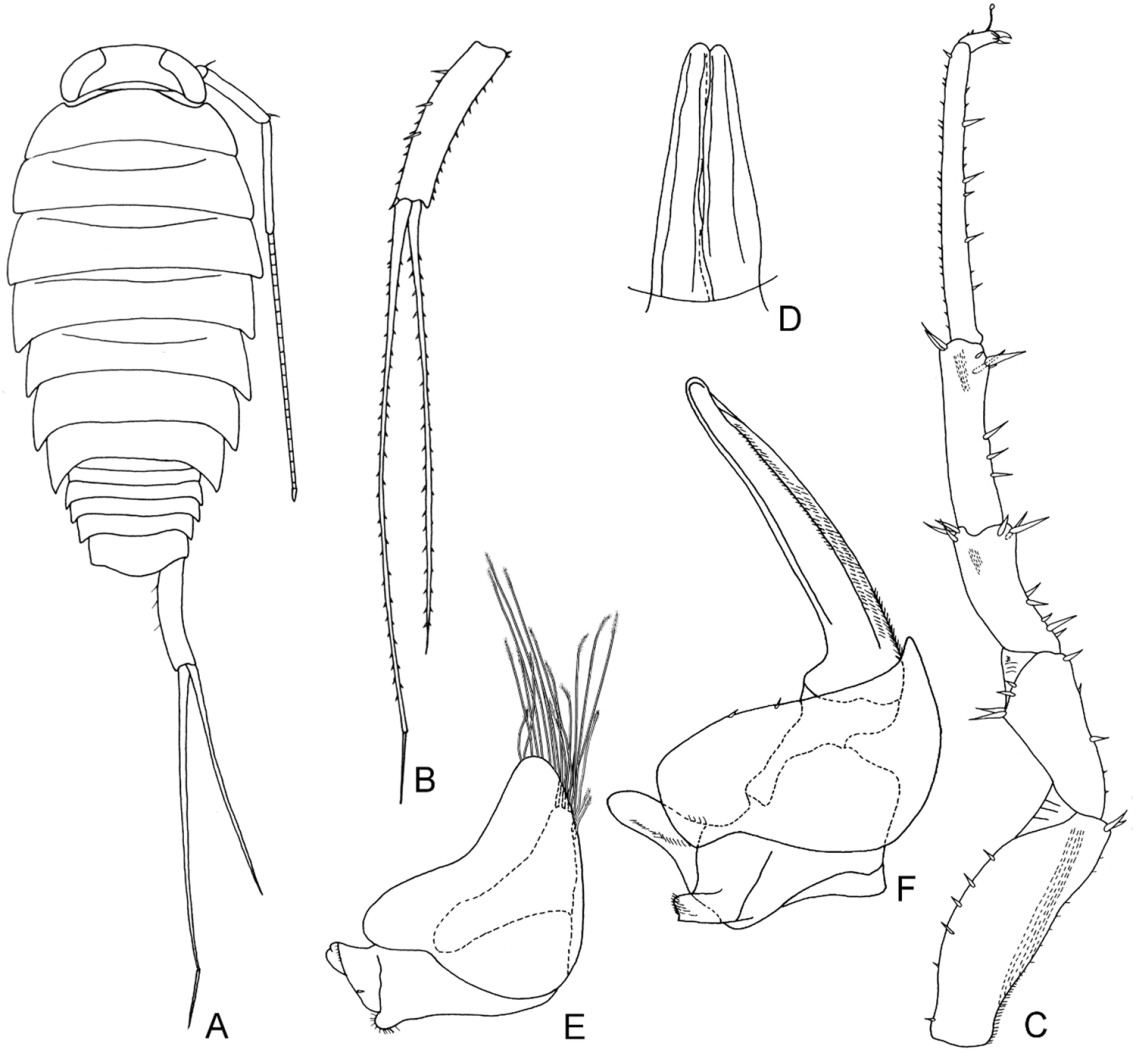
*Ligia italica* Fabricius, 1798 from Giannutri Island, Tuscany, Italy, ♀: (**A**) adult specimen, dorsal; (**B**) uropod. ♂: (**C**) pereopod 7; (**D**) genital papilla; (**E**) pleopod 1; (**F**) pleopod 2. Figures drawn by Taiti using the method by Montesanto^52,53^.

**Figure 2:**
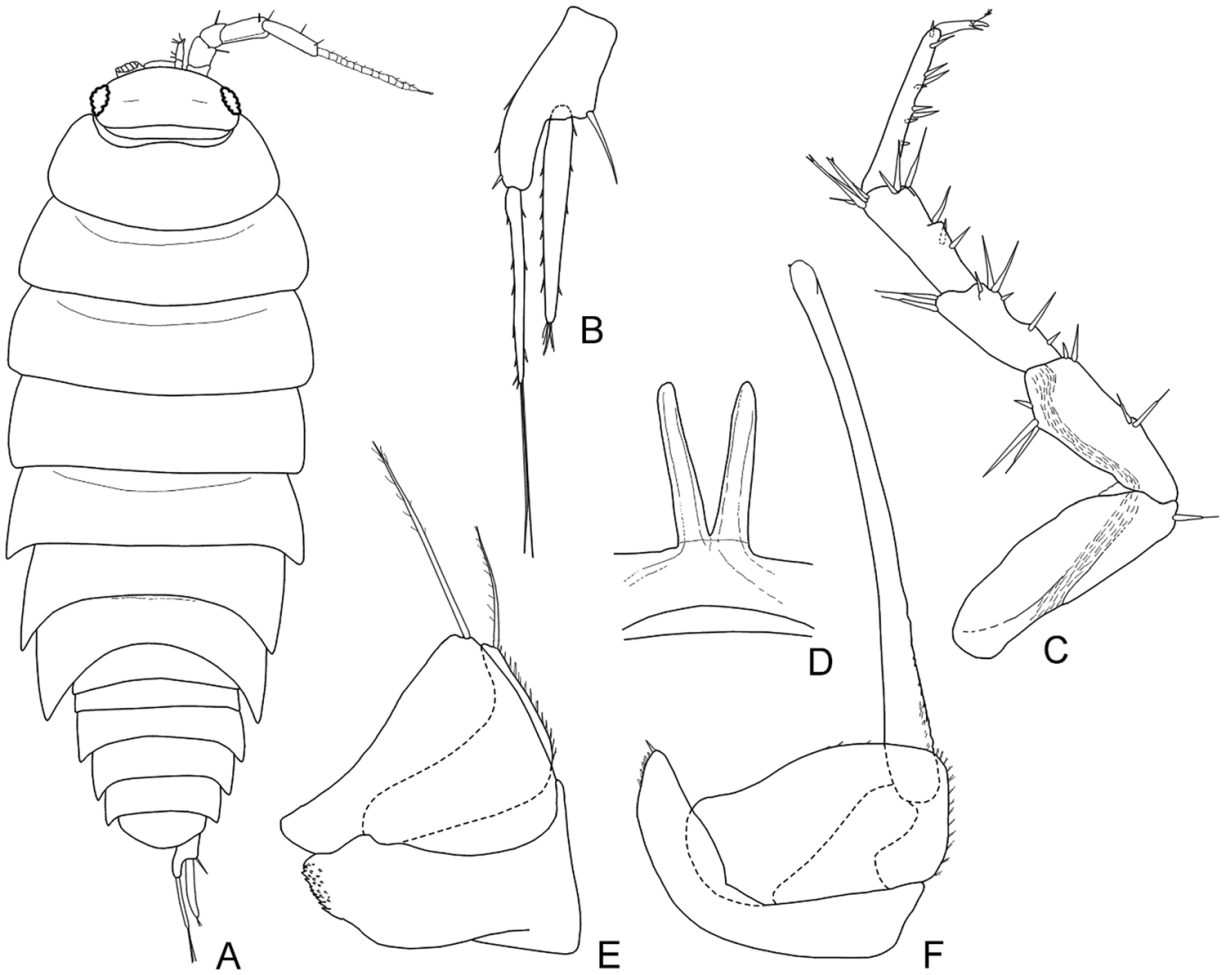
*Ligidium germanicum* Verhoeff, 1901 from Cardoso, Tuscany, Italy, ♀: (**A**) adult specimen, dorsal; (**B**) uropod. ♂: (**C**) pereopod 7; (**D**) genital papilla; (**E**) pleopod 1; (**F**) pleopod 2. Figures drawn by Taiti using the method by Montesanto^52,53^.

**Figure 3:**
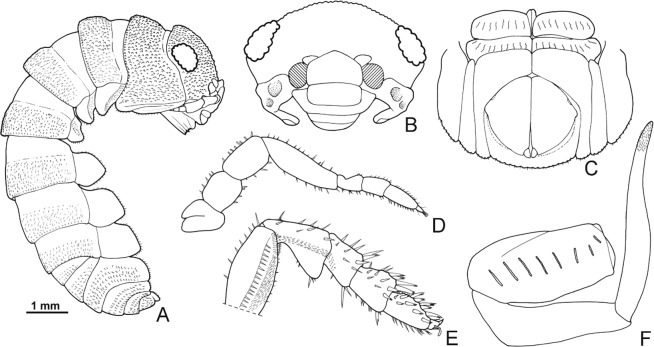
*Tylos albidus* Budde-Lund, 1885 from KudaBandos, Maldives, ♂: (**A**) adult specimen, lateral; (**B**) cephalon, frontal; (**C**) pleon and uropods, ventral; (**D**) antenna; (**E**) pereopod 7; (**F**) pleopod 2. Figures from Taiti^54^.

**Figure 4:**
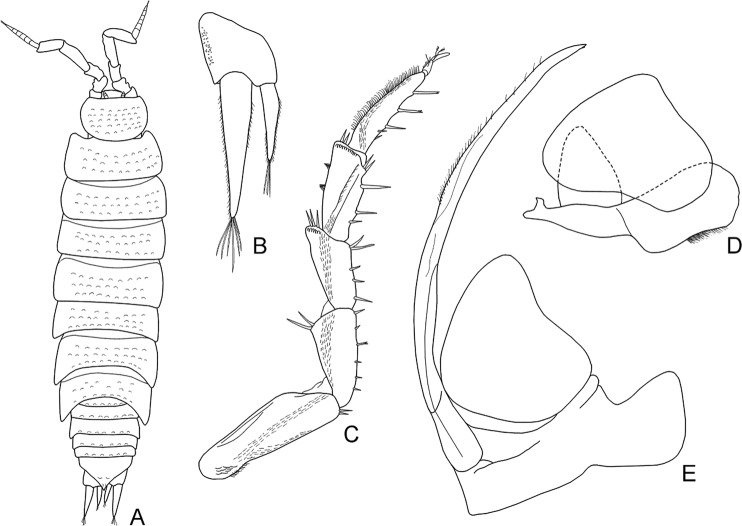
*Mesoniscus alpicola* (Heller, 1858) from San Martino cave, Varese, Lombardy, Italy, ♂: (**A**) adult specimen, dorsal; (**B**) uropod, (**C**) pereopod 7; (**D**) pleopod 1; (**E**) pleopod 2. Figures drawn by Taiti using the method by Montesanto^52,53^.

According to current taxonomy, terrestrial isopods are divided into five main clades, with the more basal ones exhibiting behavioural, ecological and morphological traits related to aquatic environments^1,5^. The more apical clades are generally more species-rich and more diverse, reflecting acquisition of vital adaptations to terrestrial environments that allowed them to conquer a wide range of habitats^2,5,6^. According to the most widely accepted phylogeny based on morphological traits, proposed by Erhard^7^, Oniscidea are divided in five major clades based on their morphological adaptations to terrestrial life and, hence, their dependence on the aquatic environment. In more detail, Diplocheta, is the most basal clade, exhibiting a series of morphological characters that suggest the form of the possible marine ancestor^6^. The two apical sister-clades are Crinocheta and Synocheta, while Microcheta constitutes their very species-poor sister-clade and Tylida have a more basal position in-between Microcheta and the ‘less terrestrial’ basal Diplocheta. Schmidt^1^ proposed a more elaborate classification, reflecting assumed phylogenetic relationships, according to which there is a basal split into Ligiidae and Holoverticata, which in turn split into Tylidae and Orthogonopoda, which consists of *Mesoniscus* Carl, 1906 and Euoniscoidea. The latter comprises the two major clades Synocheta and Crinocheta. Some of the most important characters that differ among taxa belonging to the major basal clades of Oniscidea are shown in Figs. 1–4. In particular, Figs. 1 and 2 show characters of the major genera in Ligiidae, Fig. 3 shows one of the two genera in Tylidae, and Fig. 4 shows the only genus in Microcheta.

The phylogenetic position of Oniscidea within Isopoda has been based mainly on morphological characters with controversial results so far, even regarding their monophyly^5,8–10^. Brusca and Wilson^10^ proposed Calabozoidea as sister group of Oniscidea, while Tabacaru and Danielopol^11^ suggested Valvifera as the sister group. Dreyer and Wägele^12^ conducted a molecular phylogeny based on one nuclear DNA marker and proposed Scutocoxifera as a monophyletic clade including Oniscidea, Valvifera, Sphaeromatidea, Anthuridea and Cymothoida, with Oniscidea as the basal clade in the group.

The monophyly of Oniscidea has been supported by several, presumably well-documented synapomorphies^1,5,7,11,13,14^. The most important of these are: (1) the water conducting system, formed by scales on the ventral side of coxal plates, (2) the relatively short pleotelson, (3) an antennula with less than four articles, (4) the absence of the mandibular palp, (5) the occurrence of setae on the mandible in two groups, one growing on the *lacinia mobilis*, (6) the presence of only one moveable sclerite on the basis of the second maxilla, (7) a single coxal sclerite on the maxilliped, (8) a non-subchelate first pereopod, (9) a sexually-dimorphic first pleopod, and (10) the occurrence of scale-setae on tergites. Nevertheless, Michel-Salzat and Bouchon^15^, based on mtDNA markers and a similarity-based tree, suggested that *Ligia* Fabricious, 1798 (Diplocheta, Ligiidae) is closer to Valvifera, and *Tylos* Audouin, 1826 (Tylida) to Sphaeromatidea than to the other Oniscidea. A more recent study by Lins *et al*.^16^ arrived at similar conclusions, using a Bayesian Inference approach in the analysis of two datasets, one consisting of 18 s and 28 s rRNA and COI sequences, and one comprising 13 mitochondrial protein-coding genes, but for a limited number of specimens. In both cases, *Ligia* and Tylida (included only in the first dataset) were not included in the statistically well-supported group formed by the rest of Oniscidea. Unlike Tylida, represented by *Tylos* and *Helleria* Ebner, 1868, whose close evolutionary relationship has strong statistical support, the monophyly of Ligiidae is not well supported.

Furthermore, based solely on morphological characters, Vandel^17,18^ had proposed a repetitive invasion of isopods from aquatic to terrestrial environments that happened at least three times. More specifically, Vandel^17,18^ had suggested that terrestrial isopods should be divided into three lineages: (i) “Tylienne” (=Tylida - restricted to coastal areas), (ii) “Trichoniscienne” (=Trichoniscidae + Styloniscidae? - restricted to humid micro-habitats), and (iii) “Ligienne”, which includes all remaining taxa that originated from an ancestor similar to the modern amphibious genus *Ligia*. The hypothesis that Tylida is more closely related to aquatic ancestors than the rest of Oniscidea was also supported by Tabacaru and Danielopol^11^. Nevertheless, this hypothesis was based exclusively on a single morphological character (i.e., clearly distinct coxal plates from tergites, see Fig. 3A). Overall, it is widely believed that the transition from marine to terrestrial environment was direct, without an intermediate freshwater stage^19–21^.

Herein, we aim to investigate the phylogenetic relationships among major clades of Oniscidea, in order to evaluate the validity of current taxonomy and discuss issues related to the origins of terrestrial isopods. For this purpose, in addition to the traditionally used 18 s and 28 s ribosomal RNA genes, we also targeted the highly conserved, thus suitable for the resolution of deep phylogenies, protein-coding Sodium-Potassium Pump (NAK) and Phosphoenolpyruvate Carboxykinase (PEPCK)^22–24^ genes.

## Results

Extracted DNA concentration was >15 ng/μl in all cases, with the A260/A280 purity rate over 1.5. Attempts to amplify and sequence all targeted loci were successful for almost all samples. The final compiled aligned dataset after Gblocks treatment consisted of 1,984 base pairs (bp). The initial alignment lengths and numbers of conserved, variable and parsimony-informative sites are shown in Table 1 for all sequenced loci separately. Among the tested models, the highest Akaike weight values, indicating the best fit to data, were exhibited by TIM2ef + I + G for 18 s, TIM3 + G for 28 s, TIM2 + I + G for NAK, and GTR + G for PEPCK.

**Table 1:** Aligned bases length, before and after GBlocks treatment (for ribosomal genes), conserved, variable and parsimony-informative sites for all genes used in this study.

Gene	Alignment length (bp)		Conserved sites	Variable sites	Parsimony informative sites
	Before Gblocks Treatment	After Gblocks Treatment			
18 s	1031	532	373	479	287
28 s	1857	297	221	1,055	666
NAK	639	—	303	256	639
PEPCK	516	—	247	261	214

**Figure 5:**
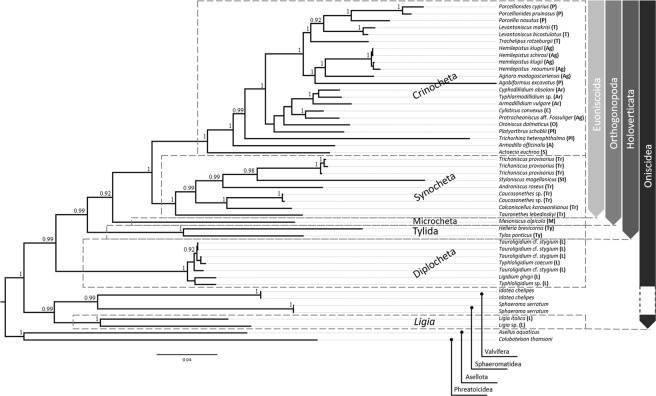
Fifty percent majority-rule consensus tree of the Bayesian Inference (BI) analysis constructed using 18 s,28 s, NAK and PECK markers. Posterior probabilities (>90) are given above nodes. Letters within brackets at tip labels indicate the family of each specimen. L: Ligiidae, Ty: Tylidae, M: Mesoniscidae, Tr: Trichoniscidae, St: Styloniscidae, Pl: Platyarthridae, C: Cylisticidae, O: Oniscidae, S: Scyphacidae, Ag: Agnaridae, T: Trachelipodidae, P: Porcellionidae, Ar: Armadillidiidae, A: Armadillidae.

Prior to calculation of genetic divergence, available sequences were grouped at the suborder level and those of Oniscidea were further grouped into the five known major subclades. *Ligia* specimens were grouped separately from the rest of the Diplocheta, as they appear to form a separate clade on the produced phylogenetic tree (Fig. 5). Genetic distances between examined taxa appeared to be constantly higher for ribosomal genes compared to the protein-coding ones. Genetic variation ranged between 6.6–30.2% in the case of 18 s, 33.3–71.6% for 28 s, 16.7–30.6% for NAK and 19.3–29.5% for PEPCK. The minimum and maximum genetic divergence values were not constantly found between the same groups for all genetic markers. More specifically, the maximum genetic distance was found between Tylida-Crinocheta, Sphaeromatidae-Crinocheta, Asellota-Valvifera and Asellota-Crinocheta, whereas the minimum values were identified between Asellota-Phreatoicidea, Tylida-*Mesoniscus*, *Ligia-*Sphaeromatidae and Valvifera-‘Diplocheta’ (excluding *Ligia*) in the case of 18 s, 28 s, NAK and PEPCK genes, respectively. All within- and between-group p-distances are given in Supplementary Material.

The Bayesian Inference (BI) and Maximum Likelihood (ML) trees exhibited largely congruent topologies. Nevertheless, in some cases, high BI posterior probabilities did not coincide with high ML bootstrap values (>80). This can be attributed to the fact that, in contrast to BI, the ML method implemented in available softwares (e.g. RAxML, PhyML, IQ-TREE) perceives gaps (−) and missing data (given as N or? in DNA alignments) as unknown characters that do not provide additional information for the resolution of phylogenetic relationships. Two out of four targeted loci are coding rRNAs whose three-dimensional structure is dependent on highly conserved regions which are interrupted by variable regions accumulating mutations, including indels. These regions are not under strong evolutionary pressure and, hence, mutations can explain the occurrence of gaps in final alignments. On the other hand, the BI approach takes into account insertion and deletion events that contain phylogenetically useful information. Therefore, only the BI tree is presented herein (Fig. 5).

Holoverticata (*sensu* Schmidt^1^) is recovered as a well-supported clade, containing the traditionally recognised sub-clade structure: Crinocheta and Synocheta form two well-supported, monophyletic sister clades, and Microcheta is the intermediate clade of these and the more basal, monophyletic Tylida. Nevertheless, Diplocheta (hence, also Ligiidae) appear to be polyphyletic, with *Ligia* being the sister taxon of Valvifera + Sphaeromatidea, and the genera *Ligidium* Brandt, 1833*, Tauroligidium* Borutzky, 1950 and *Typhloligidium* Verhoeff, 1918, traditionally grouped in Ligiidae, forming a well-supported monophyletic group, as the sister clade of Holoverticata. The monophyly of Oniscidea as currently defined is questioned, and could be saved if *Ligia* is excluded from the taxon. The basal position of *Colubotelson* Nicholls, 1944 (Phreatoicidea) and *Asellus* Geoffroy, 1762 (Asellota), as well as the statistically supported retrieval of Valvifera and Sphaeromatidae within the ‘Onisicdea’ clade, indicates the closer relationship of terrestrial isopods with these two suborders. Phylogenetic relationships inside Crinocheta also show some interesting patterns with important implications for oniscidean taxonomy. Porcellionidae form a well-supported clade with Trachelipodidae and part of Agnaridae (as the latter appear to be polyphyletic), while Armadillidiidae, traditionally considered sister-group of the Porcellionidae, is grouped with representatives of other families (e.g., Cylisticidae and part of Agnaridae). Also, *Platyarthrus* Brandt, 1833 and *Trichorhina* Budde-Lund, 1908, presently included in the family Platyarthridae, do not seem to be related, and the representative of the most diverse family Armadillidae appears in a more basal position within Crinocheta.

Within Synocheta, the monophyly of Trichoniscidae is not supported, as *Styloniscus* Dana, 1852, type-genus of Styloniscidae, seems to fall within the former. Moreover, no support for the monophyly of the subfamilies Trichoniscinae and Haplophthalminae could be found.

## Discussion

This is the first time that nuclear protein-coding genes are used to resolve phylogenetic relationships among major groups of Oniscidea. The fact that this study is so far the only one that produced a fully resolved and robust molecular phylogeny of all five major oniscidean clades, proves the advantages of using these markers. NAK has been used before^25^ in terrestrial isopod phylogenetics, but at a lower taxonomic level. Of course, given the depth of phylogeny attempted herein, the use of mitochondrial genes, with their high mutation rates and, hence, saturation effects, is not appropriate^26^. Also, the use of untreated nuclear ribosomal genes sequences, such as of 18 s and/or 28 s, might have led to biased or insufficiently supported results, as they contain regions that evolve at very different rates. Gblocks treatment was recruited to overcome possible issues that may arise due to the properties of these regions. Herein, we managed to produce a robust and sufficiently inclusive phylogeny of terrestrial isopods using a more reliable data set of nuclear DNA markers. This phylogeny has important implications for oniscidean systematics, as it undermines the validity of several morphological characters traditionally used in terrestrial isopod taxonomy. The transition of isopods from the marine to the terrestrial environment might also need to be revisited in light of the new evidence.

A number of unique adaptations to terrestrial life have led authors to assume that Oniscidea underwent only one transition from marine to land^2,6,27^. However, the low number of studies using molecular data in the past failed to confirm the monophyly of Oniscidea^15,16^, but also failed to provide a consistent phylogenetic pattern^28,29^. According to the results of our analysis, the monophyly of Oniscidea, as currently defined, is not supported, since the genus *Ligia*, generally considered as con-familiar with *Ligidium* and a small number of other related taxa, none of which exploit littoral environments, appears to be a closer relative of a group of marine isopods, such as the Valvifera and Sphaeromatidae. The monophyly of Oniscidea could be saved if *Ligia* is excluded. The assumed synapomorphies of ‘Ligiidae’, such as the residual maxillipedal segment at the back of the cephalon, are rather symplesiomorphies, as has been previously suspected^1^. *Ligidium* and related genera of the polyphyletic family Ligiidae could be assigned to a new family (we propose Ligidiidae, from the most speciose genus *Ligidium*) that can be more safely defined by more reliable synapomorphies, such as the shape of the uropods with the endopod inserted distally compared to the exopod (cf. Figs. 1B and 2B). The genus *Ligidioides* Wahrberg, 1922 (not included in our analysis) has a uropod more similar to that of *Ligia*, i.e., with the insertions of the endopod and exopod at the same level^30^, and might remain in the family Ligiidae, but this has to be investigated by a future molecular analysis that also includes this genus. Lins *et al*.^16^ came to similar conclusions regarding the relationships of *Ligia* with marine taxa, but these authors did not include other Ligiidae in their analysis, so they could not discuss the monophyly of the family. A common evolutionary history of the mitochondrial genomes of *Ligia* and *Idotea* Fabricius, 1798 was highlighted also by Kilpert and Podsiadlowski^31^. The high genetic divergence between *Ligia* and *Ligidium* was also evident from their distant position in the phenetic tree presented by Michel-Salzat & Bouchon^15^. Our findings are in agreement with all of these studies, a fact that further corroborates our hypothesis.

In view of the new phylogeny, the critical question regarding the transition from the marine environment to land should be addressed by taking into account the ecology of species in the major clades and, most importantly, the fact that the relevant event(s) happened sometime in the middle or even lower Mesozoic^27^, so that a large number of crucial forms might have been extinct without leaving any fossils of ancestral lineages. In fact, the oldest fossil Oniscidea are much younger and consist of highly derived forms^32^, while coastal marine or amphibious forms of animals that do not have hard skeletons, shells or teeth, are rarely fossilized anyway.

Considering that: (a) the most basal clade (Diplocheta, excluding *Ligia*) consists of freshwater-related taxa, (b) the subsequent clade (Tylida) includes taxa mostly living along marine coasts (even though the genus *Helleria* is fully terrestrial), and with a divergent morphology compared to other Oniscidea (at least regarding the form of cephalon, the distinct epimera on most thoracic segments, and the unique type of respiratory structures on pleopods, not connected to those of other taxa, see Fig. 3), and (c) Microcheta are fully terrestrial (albeit dependent on very high humidity) and they exhibit an overall morphology closer to that of the more derived Oniscidea (see Fig. 4), one might consider revisiting scenarios regarding the transition of isopods form the marine environment to land. Even though most *Ligia* species are amphibious, there are some species that live inland^33–36^. This means that we might envision a similar but independent transition that led to the common ancestor of ‘Ligidiidae’, given that this group consists today of species mostly living in close connection to freshwater. On the other hand, Tylidae might represent another transition, since they exhibit many characters that are difficult to recreate via a plausible transformation series from Diplocheta-type characters (cf. Figs. 1, 2 and 3). If this proves true, the next clade, Microcheta, which is basal to all Orthogonopoda, connected to very humid, freshwater-related habitats and with a more differentiated morphology than Tylida in many characters (cf. Figs. 3 and 4), would represent a third invasion to land, maybe using a freshwater path. Of course, this would undermine the actual monophyly of Oniscidea.

On the basis of current evidence, this is only a tentative hypothesis that has to be evaluated through careful elaboration of physiological traits and, hopefully, further fossil findings. Obviously, the very old origins of the Oniscidea^27^, coupled with the difficulty of fossilization of these organisms, might have led to the permanent loss of crucial information from several basal clades representing possible direct ancestors of terrestrial forms. The phylogenetic reconstruction based on modern forms cannot recover such extinct clades, except in the case of some exceptional, but highly unlikely, fossils being found in the future.

The monophyly of Crinocheta and Synocheta seems to be unambiguous. The hypothesis by Tabacaru and Danielopol^11^ that Synocheta is a sister taxon with Mesoniscidae cannot be supported. The phylogenetic relationships inside the two major clades reveal that certain morphological characters that have been considered important in oniscidean taxonomy, such as the type and form of pleopodal lungs, the ornamentation of tergites or the shape of uropods, might not be very useful. In particular, Porcellionidae and Armadillidiidae, even though they seem to share a similar type of pleopodal lung, at least in comparison with that in Trachelipodidae, appear to belong to distant clades; the former related to Trachelipodidae and part of Agnaridae (the monophyly of which is not supported), and the latter to Cylisticidae and other families. This is in agreement with the recent findings by Dimitriou *et al*.^25^. In turn, Cylisticidae appears to be closer to Armadillidiidae, even though they have styliform uropods. Within Synocheta, the traditional distinction between Trichoniscinae and Haplophthalminae, based largely on the presence of ornamentation on tergites, does not seem to be supported since *Calconiscellus* Verhoeff, 1927, a member of Haplophthalminae, appears to be the sister-taxon of *Caucasonethes* Verhoeff, 1932 and nested within other genera of Trichoniscinae. Furthermore, the status of Styloniscidae as a separate family from Trichoniscidae is also undermined. More detailed analyses, using more extensive taxonomic sampling inside these clades, are necessary to clarify these issues.

The closer relationship of terrestrial isopods with Valvifera and Sphaeromatidae than with Asellota or Phreatocidea, revealed by our analysis, agrees with the hypothesis of Brusca and Wilson^10^.

In conclusion, Oniscidea should not be considered monophyletic. Systematics in this very old group, which presents an amazing case of animal invasions to land, are in urgent need of extensive revision, taking into account robust molecular evidence. New techniques, such as whole genome sequencing, transcriptomics and ultra-conserved elements, should be applied to the whole range of terrestrial isopod taxa, in order to resolve the complete phylogenetic history of the group and shed light on crucial questions regarding the evolution of terrestriality in this taxon. Modern terrestrial isopoda is probably the only animal taxonomic group lower than Class that includes representatives of most steps of the transition from aquatic environments to almost all terrestrial environments, despite the presumed large number of extinct forms^37^. Furthermore, considering the fact that these animals have evolved structures analogous to the complex organs of terrestrial vertebrates, such as lungs (pleopodal lungs) and the placenta^4^ (marsupial, egg-feeding ‘cotelydons’), a detailed phylogenetic reconstruction can provide invaluable information on many exciting aspects of evolutionary biology, but also physiology, behaviour, ecology, and several other fields.

## Methods

### Sample collection

Using both field collecting, deposited and loaned material, we compiled a data set including 34 Oniscidea species, representing 30 genera and 14 families. Moreover, non-Oniscidea specimens of Valvifera (*Idotea*), Sphaeromatidea (*Sphaeroma* Bosc, 1801) and Asellota (*Asellus*) were also included. Colleagues that kindly sent us material are mentioned in the Acknowledgements. Freshly collected specimens, as well as the majority of available museum specimens were placed in 96% ethanol until further laboratory procedures, but we also managed to retrieve genetic data from specimens preserved in 70% alcohol for a relatively long period. Detailed information about specimens is given in Table 2.

**Table 2:** Species, locality of origin and GenBank accession numbers of individuals used in the molecular phylogenetic analyses.

Species	Family	Suborder	Section	Origin	Genes/Acc. number			
					18 s	28 s	NAK	PEPCK
*Ligia italica* Fabricius, 1798	Ligiidae	Oniscidea	Diplocheta	Cyprus	MN171516	MN174838	MN234250	MN234312
*Ligia oceanica* Linnaeus, 1767	Ligiidae	Oniscidea	Diplocheta	Galicia (Spain)	AF255698	—	—	—
*Ligia hawaiensis* Dana, 1853	Ligiidae	Oniscidea	Diplocheta	Hawaii	—	KF546702	—	—
*Ligia exotica* Roux, 1828	Ligiidae	Oniscidea	Diplocheta	Kanagawa (Japan)	—	—	MG676443	—
*Ligia exotica* Roux, 1828	Ligiidae	Oniscidea	Diplocheta	China	—	—	—	KF002742
*Ligidium ghigii* Arcangeli, 1928	Ligiidae	Oniscidea	Diplocheta	Greece	MN171506	MN174818	MN234284	MN234303
*Tauroligidium* cf*. stygium* Borutzky, 1950	Ligiidae	Oniscidea	Diplocheta	Crimea	MN171509	MN174821	MN234255	MN234307
					MN171507	—	MN234256	MN234306
					—	MN174820	MN234270	MN234305
					—	MN174819	MN234271	MN234304
*Typhloligidium coecum* (Carl, 1904)	Ligiidae	Oniscidea	Diplocheta	Crimea	M171508	MN174822	—	MN234308
*Typhloligidium coecum*	Ligiidae	Oniscidea	Diplocheta	Caucasus	MN171510	MN174823	MN234251	MN234309
*Helleria brevicornis* Ebner, 1868	Tylidae	Oniscidea	Tylida	France	MN171518	MN174843	MN234285	MN234320
*Tylos ponticus* Grebnicki, 1874	Tylidae	Oniscidea	Tylida	Cyprus	MN171519	MN174844	MN234265	—
*Mesoniscus alpicola* (Heller, 1858)	Mesoniscidae	Oniscidea	Microcheta	Italy	MN171513	MN174829	MN234249	MN234321
*Styloniscus magellanicus* Dana, 1853	Styloniscidae	Oniscidea	Synocheta	Argentina	MN171512	MN174832	—	—
*Androniscus roseus* (C. Koch, 1838)	Trichoniscidae	Oniscidea	Synocheta	The Netherlands	MN171501	MN174824	MN234283	MN234313
*Calconiscellus karawankianus* (Verhoeff, 1908)	Trichoniscidae	Oniscidea	Synocheta	Croatia	—	MN174827	MN234277	MN234319
*Caucasonethes* sp.	Trichoniscidae	Oniscidea	Synocheta	Caucasus	—	MN174826	MN234268	MN234318
					—	MN174825	MN234269	MN234317
*Tauronethes lebedinskyi* Borutzky, 1949	Trichoniscidae	Oniscidea	Synocheta	Crimea	MN171505	MN174831	MN234272	MN234322
*Trichoniscus provisorius* Racovitza, 1908	Trichoniscidae	Oniscidea	Synocheta	Cyprus	MN171502	MN174834	MN234259	MN234314
					MN171503	MN174836	MN234253	MN234315
					MN171504	MN174835	MN234286	MN234316
*Agnara madagascariensis* (Budde—Lund, 1885)	Agnaridae	Oniscidea	Crinocheta	U.A.Emirates	MG887977	MG888003	MG887924	MN234325
*Hemilepistus klugii* (Brandt, 1833)	Agnaridae	Oniscidea	Crinocheta	Iran	MG887978	MG888011	MG887926	—
*Hemilepistus schirasi* Lincoln, 1970	Agnaridae	Oniscidea	Crinocheta	Iran	MG887979	MG888012	MG887927	—
*Hemilepistus reaumurii* (Milne-Edwards, 1840)	Agnaridae	Oniscidea	Crinocheta	Tunisia	MN171500	MN174828	MN234258	—
*Protracheoniscus* aff. *fossuliger* (Verhoeff, 1901)	Agnaridae	Oniscidea	Crinocheta	Greece	MN171494	MN174817	MN234281	MN234292
*Armadillo officinalis* Dumeril, 1816	Armadillidae	Oniscidea	Crinocheta	Cyprus	MN171498	MN174812	MN234252	—
*Armadillidium vulgare* (Latreille, 1804)	Armadillidiidae	Oniscidea	Crinocheta	Cyprus	MN171495	MN174837	—	MN234299
*Cyphodillidium absoloni* (Strouhal, 1934)	Armadillidiidae	Oniscidea	Crinocheta	Croatia	—	MN174814	MN234276	MN234295
*Typhlarmadillidium* sp.	Armadillidiidae	Oniscidea	Crinocheta	Croatia	—	MN174815	MN234273	MN234294
*Cylisticus convexus* (De Geer, 1778)	Cylisticidae	Oniscidea	Crinocheta	Greece	MN171493	MN174813	MN234280	MN234293
*Oroniscus dalmaticus* Strouhal, 1937	Oniscidae	Oniscidea	Crinocheta	Croatia		MN174816	MN234274	MN234297
*Platyarthrus schoblii* Budde-Lund, 1885	Platyarthridae	Oniscidea	Crinocheta	Cyprus	MN171492	MN174833	MN234254	MN234298
*Trichorhina heterophthalma* Lemos de Castro, 1964	Platyarthridae	Oniscidea	Crinocheta	The Netherlands (greenhouse)	MN171496	MN174845	MN234282	MN234300
*Agabiformius excavatus* Verhoeff, 1941	Porcellionidae	Oniscidea	Crinocheta	Cyprus	MG887969	MG888009	MG887921	—
*Porcellio nasutus* Strouhal, 1936	Porcellionidae	Oniscidea	Crinocheta	Cyprus	MG887980	MG887999	MG887911	—
*Porcellionides cyprius* (Strouhal, 1968)	Porcellionidae	Oniscidea	Crinocheta	Cyprus	MN171488	MN174808	MN234278	MN234287
*Porcellionides pruinosus* (Brandt, 1833)	Porcellionidae	Oniscidea	Crinocheta	Cyprus	MN171489	MN174809	MN234275	MN234288
*Actaecia euchroa* Dana, 1853	Scyphacidae	Oniscidea	Crinocheta	New Zealand	MG887985	MG888007	MG887930	MN234324
*Levantoniscus makrisi* Cardoso, Taiti and Sfenthourakis, 2015	Trachelipodidae	Oniscidea	Crinocheta	Cyprus	MN171490	MN174810	MN234260	MN234289
*Levantoniscus bicostulatus* Cardoso, Taiti and Sfenthourakis, 2015	Trachelipodidae	Oniscidea	Crinocheta	Cyprus	MN171491	MN174811	MN234257	MN234290
*Trachelipus ratzeburgii* (Brandt, 1833)	Trachelipodidae	Oniscidea	Crinocheta	Germany	MN171497	MN174830	MN234279	MN234291
*Asellus aquaticus* (Linnaeus, 1758).	Asellidae	Asellota	—	Greece	MN171511	MN174846	MN234267	MN234323
*Colubotelson thomsoni* Nicholls, 1944	Phreatoicidae	Phreatoicidea	—	Tasmania	AF255703	AF169711	—	—
*Sphaeroma serratum* (Fabricius, 1787)	Sphaeromatidae	Sphaeromatidea	—	Italy	MN171520	MN174842	MN234262	MN234301
					MN171517	MN174841	MN234261	MN234302
*Idotea chelipes* (Pallas, 1766)	Idoteidae	Valvifera	—	Italy	MN171515	MN174840	MN234263	MN234311
					MN171514	MN174839	MN234264	MN234310

(√ will be replaced with accession numbers when available).

### Amplification of targeted loci

Total genomic DNA was extracted from available specimens using a DNeasy Blood and Tissue Kit (Qiagen, Hilden, Germany), following the manufacturer’s proposed protocol. Quality and quantity control of extracted DNA was performed with NanoDrop 2000/200c (Thermo Fisher Scientific Inc., USA). The final concentration was measured in ng /μl and purity was verified with A260/A280nm absorption ratio.

The non-coding nuclear genetic markers 18 s and 28 s, and the protein-coding Sodium-Potassium Pump (NAK) and Phosphoenolpyruvate Carboxykinase (PEPCK) genetic loci were targeted with common PCR procedures using gene specific primers. Desired regions were successfully amplified using 18Aimod/700 R primer pair for 18s^38^, 28sa/28 sb for 28s^39^, NAK for-b/NAK rev 2 or NAK for-b/NAK 638 R for NAK^24,25^ and PEPCKfor/PEPCKrev^24^ and the newly designed PEPCK 545 R (5′-CCRAAGAANGGYSTCATNGC-3′) for PEPCK. All PCR reactions were carried out in a Veriti thermal cycler (Applied Biosystems, USA). Taking into account the genetically diverse samples, we used a touchdown PCR approach to eliminate aspecific products and save time, opposed to using multiple reactions, specific for different taxa. This way we managed to increase specificity, sensitivity and yield^40^. In each case, the final reaction volume was adjusted to 20 μl, including 0.5 U of Kapa *Taq* DNA Polymerase, 3 mM MgCl_2_, 1X of Kapa PCR buffer A, 0.3 mM dNTP (Kapa) 0.3 µM of each primer and >20 ng of DNA template. The reactions’ thermal profile followed Dimitriou *et al*.^25^. Amplicons were purified with a Qiaquick Purification Kit (Qiagen, Germany) following the proposed instructions. The final products were sent for sequencing of both DNA strands at Macrogen facilities (Amsterdam, The Netherlands).

### Data processing

CodonCode Aligner (v. 3.7.1; CodonCode Corp., USA) was used to manually inspect chromatograms, generate assemblages and make edits, where necessary. Our final dataset also included sequences of additional *Ligia* spp. and *Colubotelson thomsoni* Nicholls, 1944 (Phreatoicidea) retrieved from NCBI GenBank. The latter was included to serve as an additional outgroup. In the case of the genus *Ligia*, apart from the data generated in the framework of the present study, a chimeric sequence combining data from all targeted genes from the congeneric species *L. oceanica* (Linnaeus, 1767), *L. hawaiensis* (Dana, 1853) and *L. exotica* Roux, 1828 was included in our analyses. In this way, we manage to verify the phylogenetic position of the genus in the produced tree in a robust way. Accession numbers of all sequences used herein are given in Table 2. Sequences from each targeted gene were separated in different files and multiple sequence alignments were performed using MAFFT v.7^41^. MEGA v.6^42^ was used to calculate genetic distances for each alignment. Relatively longer sequences with no overlapping fragments for the majority of the samples were trimmed prior to further data elaboration.

Given that ribosomal genes consist of multiple conserved and flanking hypervariable regions, related to their functional three-dimensional structure after gene expression, alignment might be challenging^43^. In order to test the sensitivity of produced alignments and remove possible poorly aligned regions for 18 s and 28 s genes, we used Gblocks v0.91b^44^ through the Gblocks server available at http://molevol.cmima.csic.es/castresana/Gblocks_server.html. The analysis was run allowing smaller final blocks, less strict flanking and gap positions. The positive effects of removing divergent and ambiguously-aligned blocks in phylogenies are discussed by Talavera and Castresana^45^.

### Phylogenetic analyses

The optimal nucleotide substitution model for each loci was selected according to Akaike’s Information Criterion (AIC)^46^ using jModeltest v.2.1.1^47^. Phylogenetic reconstructions were conducted with BI and ML methods implemented in MRBAYES v. 3.2.6^48^ and RAxML-NG web server^49^ respectively.

The concatenated data set was fed as partition blocks to MrBayes. Bayesian Inference analysis was run with the selected model of nucleotide evolution for each gene, under the default settings for within-partition among-site rate variation, allowing rate heterogeneity between partitions. BI, applying Metropolis-coupled Markov Chain Monte Carlo algorithms, was set to run four independent times with eight chains per run for 20 million generations and a sampling frequency of 100. Stationarity and convergence among runs, were ensured by monitoring the average standard deviation of split frequencies of the four simultaneous and independent runs in MrBayes. Furthermore, likelihood values, as well as all other parameters estimated as indicators for the convergence among runs were monitored using Tracer v 1.5^50^. From the sampled trees, 10% were discarded as the burn-in phase and a 50% majority-rule consensus tree was constructed from the remaining trees in MrBayes.

Maximum Likelihood trees were constructed under the same partitioning scheme and nucleotide substitution models. The reliability was tested by bootstrapping^51^ with 1,000 replicates.

## Supplementary information

Sequence divergence

## Data Availability

Genetic data used in the present study are deposited at Genbank and publicly accessible through the provided accession numbers.
