# Supplementary material for: Genetic evidence against monophyly of Oniscidea implies a need to revise scenarios for the origin of terrestrial isopods
Source: Sci Rep. 2019 Dec 6;9:18508. doi: 10.1038/s41598-019-55071-4 (PMC6898597; doi:10.1038/s41598-019-55071-4)
Supplement: Supplementary file 1 — Sequence divergence [file 41598_2019_55071_MOESM1_ESM.docx]

**Genetic evidence against monophyly of Oniscidea implies a need to revise scenarios for the origin of terrestrial isopods**

*Andreas C. Dimitriou^1^, Stefano Taiti^2^ and Spyros Sfenthourakis^1^

1. Department of Biological Sciences, University of Cyprus, Panepistimiou Ave. 1, 2109 Aglantzia, Nicosia, Cyprus.

2. Museo di Storia Naturale, Sezione di Zoologia “La Specola”, Via Romana 17, 50125 Florence, Italy.

**Supplementary material**

**Table S1.** Percentage sequence divergence (p-distance) among the main Oniscidea clades and other isopod suborders for 18s.

| **Group** | | **1** | **2** | **3** | **4** | **5** | **6** | **7** | **8** | **9** | **10** |
| --- | --- | --- | --- | --- | --- | --- | --- | --- | --- | --- | --- |
| **1** | Crinocheta | 13.59 |  |  |  |  |  |  |  |  |  |
| **2** | Synocheta | 12.76 | 4.42 |  |  |  |  |  |  |  |  |
| **3** | Microcheta | 15.68 | 10.96 | n/c |  |  |  |  |  |  |  |
| **4** | Tylida | 30.22 | 24.74 | 28.23 | 43.40 |  |  |  |  |  |  |
| **5** | Diplocheta* | 14.75 | 8.66 | 10.70 | 25.66 | 1.25 |  |  |  |  |  |
| **6** | Sphaeromatidea | 18.25 | 15.28 | 16.29 | 29.33 | 16.30 | 0.00 |  |  |  |  |
| **7** | *Ligia* | 16.36 | 8.78 | 12.86 | 30.00 | 10.57 | 16.48 | 11.67 |  |  |  |
| **8** | Valvifera | 15.07 | 8.42 | 13.23 | 25.89 | 8.48 | 16.73 | 8.80 | 0.00 |  |  |
| **9** | Phreatoicidea | 14.69 | 9.10 | 11.84 | 26.00 | 8.38 | 14.99 | 9.54 | 9.11 | n/c |  |
| **10** | Asellota | 14.31 | 9.33 | 8.90 | 24.97 | 8.35 | 14.53 | 8.78 | 8.20 | 6.62 | n/c |

* Not including *Ligia*, treated separately based on its position in the phylogenetic tree.

**Table S2.** Percentage sequence divergence (p-distance) among the main Oniscidea clades and other isopod suborders for 28s.

| **Group** | | **1** | **2** | **3** | **4** | **5** | **6** | **7** | **8** | **9** | **10** |
| --- | --- | --- | --- | --- | --- | --- | --- | --- | --- | --- | --- |
| **1** | Crinocheta | 46.21 |  |  |  |  |  |  |  |  |  |
| **2** | Synocheta | 43.28 | 31.57 |  |  |  |  |  |  |  |  |
| **3** | Microcheta | 43.08 | 38.12 | n/c |  |  |  |  |  |  |  |
| **4** | Tylida | 42.85 | 36.71 | 32.27 | 33.78 |  |  |  |  |  |  |
| **5** | Diplocheta* | 50.19 | 48.32 | 45.11 | 42.87 | 22.44 |  |  |  |  |  |
| **6** | Sphaeromatidea | 53.35 | 50.99 | 48.07 | 46.59 | 43.55 | 0.00 |  |  |  |  |
| **7** | *Ligia* | 52.18 | 49.99 | 47.02 | 42.42 | 48.75 | 50.31 | 60.34 |  |  |  |
| **8** | Valvifera | 51.37 | 49.98 | 44.13 | 45.33 | 37.14 | 46.38 | 50.55 | 0.00 |  |  |
| **9** | Phreatoicidea | 66.70 | 65.24 | 64.93 | 66.61 | 66.47 | 70.60 | 68.82 | 66.25 | n/c |  |
| **10** | Asellota | 68.25 | 68.93 | 71.57 | 68.38 | 68.55 | 64.40 | 71.50 | 67.54 | 71.10 | n/c |

* Not including *Ligia*, treated separately based on its position in the phylogenetic tree.

**Table S3.** Percentage sequence divergence (p-distance) among the main Oniscidea clades and other isopod suborders for NAK.

| **Group** | | **1** | **2** | **3** | **4** | **5** | **6** | **7** | **8** | **9** |
| --- | --- | --- | --- | --- | --- | --- | --- | --- | --- | --- |
| **1** | Crinocheta | 9.52 |  |  |  |  |  |  |  |  |
| **2** | Synocheta | 17.88 | 12.78 |  |  |  |  |  |  |  |
| **3** | Microcheta | 20.13 | 19.29 | n/c |  |  |  |  |  |  |
| **4** | Tylida | 19.95 | 19.09 | 20.09 | 16.06 |  |  |  |  |  |
| **5** | Diplocheta* | 22.23 | 20.08 | 19.82 | 20.24 | 0.62 |  |  |  |  |
| **6** | Sphaeromatidea | 19.14 | 18.80 | 18.69 | 18.80 | 17.76 | 0.00 |  |  |  |
| **7** | *Ligia* | 21.02 | 18.81 | 19.28 | 20.04 | 21.07 | 16.71 | 15.58 |  |  |
| **8** | Valvifera | 26.89 | 0.22 | 0.22 | 0.22 | 0.22 | 0.22 | 0.22 | 0.22 |  |
| **9** | Asellota | 26.23 | 26.92 | 26.78 | 26.40 | 30.57 | 27.75 | 27.71 | 30.64 | n/c |

* Not including *Ligia*, treated separately based on its position in the phylogenetic tree.

**Table S4.** Percentage sequence divergence (p-distance) among the main Oniscidea clades and other isopod suborders for PEPCK.

| **Group** | | **1** | **2** | **3** | **4** | **5** | **6** | **7** | **8** | **9** |
| --- | --- | --- | --- | --- | --- | --- | --- | --- | --- | --- |
| **1** | Crinocheta | 9.10 |  |  |  |  |  |  |  |  |
| **2** | Synocheta | 20.56 | 15.08 |  |  |  |  |  |  |  |
| **3** | Microcheta | 23.82 | 22.62 | n/c |  |  |  |  |  |  |
| **4** | Tylida | 23.09 | 22.84 | 24.67 | n/c |  |  |  |  |  |
| **5** | Diplocheta* | 21.75 | 22.98 | 22.44 | 20.98 | 2.24 |  |  |  |  |
| **6** | Sphaeromatidea | 22.43 | 24.51 | 22.86 | 25.82 | 22.41 | 0.00 |  |  |  |
| **7** | *Ligia* | 22.29 | 23.32 | 23.44 | 23.73 | 22.71 | 21.75 | 18.76 |  |  |
| **8** | Valvifera | 22.32 | 24.68 | 24.23 | 22.35 | 19.31 | 20.04 | 20.11 | 0.00 |  |
| **9** | Asellota | 29.46 | 28.80 | 28.50 | 28.76 | 25.25 | 27.44 | 26.16 | 23.75 | n/c |

* Not including *Ligia*, treated separately based on its position in the phylogenetic tree.
